# Modelling multi-rotor UAVs swarm deployment using virtual pheromones

**DOI:** 10.1371/journal.pone.0190692

**Published:** 2018-01-25

**Authors:** Fidel Aznar, Mar Pujol, Ramón Rizo, Carlos Rizo

**Affiliations:** 1 Department of Computer Science and Artificial Intelligence, University of Alicante, San Vicente del Raspeig, Alicante, Spain; 2 Department of Architectural Constructions. University of Alicante, San Vicente del Raspeig, Alicante, Spain; Southwest University, CHINA

## Abstract

In this work, a swarm behaviour for multi-rotor Unmanned Aerial Vehicles (UAVs) deployment will be presented. The main contribution of this behaviour is the use of a virtual device for quantitative sematectonic stigmergy providing more adaptable behaviours in complex environments. It is a fault tolerant highly robust behaviour that does not require prior information of the area to be covered, or to assume the existence of any kind of information signals (GPS, mobile communication networks …), taking into account the specific features of UAVs. This behaviour will be oriented towards emergency tasks. Their main goal will be to cover an area of the environment for later creating an ad-hoc communication network, that can be used to establish communications inside this zone. Although there are several papers on robotic deployment it is more difficult to find applications with UAV systems, mainly because of the existence of various problems that must be overcome including limitations in available sensory and on-board processing capabilities and low flight endurance. In addition, those behaviours designed for UAVs often have significant limitations on their ability to be used in real tasks, because they assume specific features, not easily applicable in a general way. Firstly, in this article the characteristics of the simulation environment will be presented. Secondly, a microscopic model for deployment and creation of ad-hoc networks, that implicitly includes stigmergy features, will be shown. Then, the overall swarm behaviour will be modeled, providing a macroscopic model of this behaviour. This model can accurately predict the number of agents needed to cover an area as well as the time required for the deployment process. An experimental analysis through simulation will be carried out in order to verify our models. In this analysis the influence of both the complexity of the environment and the stigmergy system will be discussed, given the data obtained in the simulation. In addition, the macroscopic and microscopic models will be compared verifying the number of predicted individuals for each state regarding the simulation.

## Introduction

Multi-robot systems have higher flexibility, efficiency and reliability than single robots: a team of collaborative robots can accomplish a single task much faster, execute tasks beyond the limits of single robots, perform a complex task with multiple specialized simple robots rather than a super robot, and provide distributed, parallel mobile sensing and processing. Exploring an unmapped terrain by mobile robots is a fundamental and complex problem [1]. The research results have found many potential real-world applications in cleaning, mowing, search-and-rescue operation, ocean monitoring and planetary exploration [2–8].

Bio-inspired algorithms provide excellent strategies to solve the unknown terrain covering problem. The ants have simple sensor ability, limited computational power and a decentralized control system. These features make it practical to build a large number of ant-like robots to explore an unknown terrain. Several covering algorithms were developed for ant-like robot systems [9–11].

Distributed control allows each member robot to determine its motion according to the states of itself, its local environment and its interactions with nearby robots and other objects. Distributed processing largely reduces the computational and communication complexities. As a result, distributed control is highly scalable to large multi-robot systems and adaptive to unknown and dynamic environments and changes in multi-robot systems. With properly designed distributed control laws, the desired global goal of a multi-robot system can be achieved as the combined outcome of the self-deployment motion of individual robots.

Swarms of flying robots are a promising alternative to ground-based robots when searching in indoor environments with advantages such as increased speed and the ability to fly above obstacles. However, there are numerous problems that must be overcome including limitations in available sensory and on-board processing capabilities, and low flight endurance.

In this paper, a swarm behaviour for swarm deployment will be presented. This task will be carried out without any prior information about the environment, which can be structured or even unstructured. In addition we will assume that the swarm individuals will have no access to any communications network or localization service, such as GPS. In this way, these individuals must deploy in the environment their own communications network to send or receive information using the proposed sigmergy system. Our goal is that this behaviour can be used in emergency tasks, where the requirements of operation are much more restrictive. Once we discuss the microscopic behaviour of the swarm we will introduce a macroscopic model. This mathematical model predicts the number of individuals that execute each of the behaviour states, which allows us to forecast the system performance without simulating or testing the behaviour. To validate our the macroscopic behaviour, an analysis of this mathematical model versus several simulations will be discussed.

More specifically, in the next section we will deal with the state of the art of deployment behaviours for multi-robot systems in general and for multi-rotor UAVs in particular. Then, both, the platform of swarm individuals and the physical simulator used for the testing phase will be presented. We will continue by proposing a microscopic model for environment covering using UAVs. In the next section, the mathematical model capable of capturing the microscopic system operation will be formalized. In addition, we will show the microscopic behaviour in a simulated way and will compare it with our macroscopic model. We will finish by providing the conclusions of this work and some future lines.

## Related work

Multi-robot deployment has become a fundamental research topic in the field of multi-robot systems. In this section we will show a brief review of the existing types of swarm deployment and coverage of terrestrial robots. Then we will review some applications designed to be executed using aerial vehicles and comment some examples of stigmergy communication in swarm systems.

Although there are many applications related to multi-robot deployment, many of them rely on pre-established communication network or external localization service. This precludes its application to emergency tasks, where we are not able to assume the existence of communication networks or GPS. In this way, there are several studies where the external communication network is substituted by the deployment of an own network of sensor nodes or radio beacons. For example [12] uses a distributed algorithm able to guide a group of robots through a network of sensors. However this strategy and others similar ones require the prior deployment of the sensor network which could be no practical and difficult in emergency situations.

Another alternative is to include the deployment process in the behaviour. In this case the behaviour consists of two fases: beacon deployment and robot deployment. [13] deploy a network of static radio beacons forming a long range communication network that could aid robot exploration. However, such strategies cannot be used with micro or small sized UAVs (the most commonly used ones in indoor environments) because of their low load capacity and limited autonomy.

In [14, 15] a different strategy is presented, where each individual integrate a beacon, creating themselves their own communications network. However the design of this behaviour is intended for the physical characteristics of terrestrial robots *s-bot*, which makes very difficult to generalize it to other types of vehicles due to their different types of sensors and actuators.

In our case, we are interested in developing a behaviour that uses the advantages of indoor flying vehicles, compared to terrestrial ones, in emergency situations. For example, these vehicles allow to avoid many obstacles that could limit ground vehicles, besides allowing sensorial data to be obtained from a vantage point of view [16–20].

This type of devices require specific considerations and algorithms to ensure their navigability [21]. On the one hand, global localization mechanisms cannot be used, as it is common in outdoor UAVs [22, 23]. Thus, in outdoor UAVS the GPS signal is the most common localization system, which can be even adjusted by using onboard inertial sensors. Such systems cannot neither be used indoors (signal reception occlusion) nor in all outdoor locations and situations, so another approximation must be used, such as local localization.

Robot local localization commonly requires environment maps and odometry sensing [24]. Environment maps may be previously unknown and the online creation of maps requires powerful processing that may not be available on small flying robots [25]. Also, such approaches do not scale appropriately with large swarms [22]. Moreover, there exists simple relative localization applications suitable for small UAVs. For example in [26] a relative positioning sensors in reference to nearby static robots with a decentralised approach is presented.

Related to UAV deployment, we found one of the first applications presented in [27]. This application uses an predetermined communication network as a basis for guiding UAVs. Although this behaviour does not require an external communications network, it needs a pre-deployment phase, as the terrestrial application [12], which is not applicable in real emergency situations. In a later work [28] reconfigures the network of sensors to maximize system performance, but in the same way requires a group of terrestrial robots to transport the sensor network, which it is not practical for our needs.

More recently, [26, 29] presents a behaviour where a fully distributed deployment is developed while creating a communication network. This system does not require existing communication networks or global localization system. In addition, it is an effective strategy, designed to save energy when using UAVs, which is very important for increasing swarm autonomy. [26, 29] assumed that a robot can temporarily attach to the ceiling, or land on the ground for efficient surveillance over extended periods of time. However, as the authors discussed in that paper, this behaviour requires special environment features (straight walls, 90 degree angles,…) to be executed (is not functional for unstructured environments) and does not take into account uncertainty (for example the errors in sensorial readings or the robot actuators, which is very important in UAVs systems).

Using the ideas presented in [14, 15] and [26, 29, 30] an UAV swarm can establish their own communications network. This network can be used for communicating swarm tasks or even to help other robots or a human team for subsequent work [12, 14, 15, 27, 31, 32].

In this paper, we will present a behaviour inspired by the work of [14, 15] in terms of establishing their own network of communications and by [26, 29] in terms of adapting the behaviour to UAV using a power save state. However, in contrast to previous articles our distributed behaviour will be able to be executed in any environment, without limiting their specific features or its size.

For this purpose, a virtual sematectonic stigmergy system will be used. The use of this type of communication is not new in the field of swarm robotics, finding in the literature several applications using stigmergy as communication mechanism. In [33], a swarm behaviour that can develop collaborative tasks through sematectonic stigmergy is presented. [23] discuss a theoretical approach to the use of stigmergy in UAVs using stigmergic potential fields. To implement this type of communication it is assumed that we have a local positioning mechanism such as used in [34] and a efficient system of communication between individuals, as could be the use of mesh networks of XBEEs devices [35].

We want to underline that the probabilistic microscopic model and the stigmergy system that will presented here take into account the inherent uncertainty of the real robotic systems, which do not occur in other approaches that assume ideal worlds such as [26, 29, 30].

## Platform

Special considerations are required for a physical platform that will host a behavior demanding the deployment of a group of indoor drones. The most important UAVs features and the simulator that enables realistic testing of our behavior are described below.

Indoor flights usually require smaller drones than outdoors ones. The size and weight of these devices is directly related to their load capacity and their autonomy. We have estimated that the maximum size of multi-rotor UAV will be of half metre radius so that it lets enough room for maneuver.

Thus, micro and minidrones, or even large enough UAVs as to load several sensors and to conduct outdoor flights safely are covered here. A maximum speed of 2m/s is assumed. From our point of view this provides sufficient safety margins for indoor environments. These multi-rotor UAVs must have a mechanism that allows them to detect obstacles in its flying plane. There are several alternatives that fulfill this task in the market, varying from low cost ultrasonic sonar to more advanced and accurate small LIDARs sensors.

Our approach offers the innovative aspect of using stigmergy and hence virtual pheromones in our behavior. There are several studies that use such strategies simulating single transceivers mounted on the top of each robot. A system allowing: local positioning of several drones, and the transmission and later peer-to-peer storage is needed for the development of this behaviour. Using XBEE devices to network peer-to-peer communication can provide a simple and inexpensive way to implement this requirements.

UAVs launching will occur progressively over time from the same launching point. This will simplify the arrangements before take off and will facilitate its application in emergency tasks, where a specific launching point could be a requirement. Each UAV will be identified by a sole number, starting with the lowest number for the UAV first launched. As will be considered in the next section, this identification number will vein order to adjust the intensity of pheromones emission in the behaviour.

It is also important to specify how the percentage of coverage achieved by the swarm will be calculated. We propose the use of two metrics. We consider that each drone should have a fixed visualization area. This area will define which things of the environment are being perceived given the current position of the drone. For the first metric we have considered that once the perception area of a drone has passed through a certain place, this place has been covered. Thus, an area will be considered to have been covered when the area of detection of all the drones have covered the entire area at some time. On the other hand, the second metric assumes that the drones cannot store information from the environment so it will be the final configuration of the swarm what determines the covered area due to the limited perception range of the vehicles from their final position.

As it will be seen in next section, the radius for pheromones emission must be established in order to create a sparse or dense robot deployment and therefore, a more or less dense communication network. These networks will be created by using an special behaviour state, called beacon, where the robots must land and become part of the communication network and the stigmergy system of the swarm.

Modelling is a method used in many research fields, to better understand the internals of the investigated system, that has several advantages for swarm-robotics. The existence of possible risks for the robots and the limited power of the robots require a human observer to follow the experiments and do some house keeping works periodically. The time spent on these experiments and possible risk of losing the robots, even if a human observer exists, become a bottleneck when several experiments are needed to validate the results of the studies. To eliminate these problems it is safer and easier to model the experiments and simulate them on computers. Another importance of modelling for swarm robotic studies appears when the scalability of the experiments are tried to be tested. Most of the time, scalability requires to test the control algorithms on more than hundreds of robots *which is cost prohibitive* [36].

In addition, when designing swarm-control mechanisms, researchers and engineers are faced with the challenge to develop a set of rules at the individual (microscopic) level such that a desired behaviour at the group (macroscopic) level is achieved. This is a very difficult task since there is no general systematic way to devise individual behaviours that reliably achieve a desired group behaviour. Thus design choices can usually only be tested in experiments or simulations [37].

A swarm simulator based on [38] library has been developed in order to illustrate the experimental section of this paper. A specific physical layer for UAVs vehicles simulation has also been added. A realistic approach to multi-rotor UAVs dynamics has been considered taking also into account the errors of the actuators, sensors and lastly the inherent uncertainty of such systems altogether.

This simulator provides a unique mechanism that analyzes the performance of our behaviors on large groups of UAVs with different types of scenarios. In addition, another two items have been developed: on the one hand, a virtual system of the quantitative sematectonic pheromones. On the other hand, a network of ultrasonic sensors used for detection of other drones or surrounding walls. We have tried to be as faithful as possible to the final physical implementation.

## Proposed microscopic behaviour and virtual stigmergy sensor for multi-robot deployment

In this section, a microscopic covering behaviour for the creation of an ad-hoc communication network among all the members of a swarm will be presented. In addition an stigmergy sensor will be described taking into account the main features of the proposed behaviour. We assume UAVs that have a limited computing capacity, where a desired goal is the miniaturization of these devices and therefore the integration of the behaviour within the stabilization of the aircraft.

Thus, it seems reasonable to use the shortest CPU time possible in order to provide more room to the aircraft stabilization and other low and medium level routines that become crucial in some aircrafts, such as multi-rotor systems.

Moreover, this swarm individuals should always maintain connectivity. One of their missions is to create a network of communication between them, allowing thus to extract the required data from a specific point of the environment. It is therefore essential that no single individual remains isolated and therefore our behaviour will take measures to prevent it.

As it has been said before, a part of this microscopic behaviour is inspired by the one presented in [26, 29], where swarm individuals could stay in two different states: in flight or landed. In UAVs system autonomy is a significant limitation. The use of these two states is stablished when aiming to provide an energy saving mechanism while individuals continue as part of the system without navigability requirements.

Initially, our behaviour will starts with an agent in *beacon* (landing) state, which indicates the entry point to explore the environment. Then, an aircraft in *wander* (flying) state will be added at regular intervals. Every aircraft will have an only numeric identifier, that will be incremented for each added individual. The first mission of this behaviour is to explore the environment. Thus, the last added individual drone will be flying (*wander* state), exploring the environment by using its obstacle sensors following a probabilistic flight direction until the signal strength of the stigmergy sensor (for example using the Received Signal Strength Indication, RSSI of the receiving device) will decrease by a certain threshold, limiting the guaranteed communication distance to a maximum of li∈R meters. In this case, the individual will proceed to land and change its state to *beacon*.

In *beacon* state, the vehicle is in saving battery mode (the battery consumed when it is landed is negligible compared when it is in flight). Its mission is twofold: firstly it must put forward any signal received from a swarm member, thus establishing a communication network where each individual can send, receive and route information. This behaviour is necessary in order to send information from a control point, to develop the virtual stigmergy sensor or to obtain information regarding the environment covering. Therefore, vehicles in *beacon* state have an important role in our stigmergy implementation.

There are several types of stigmergy in nature that enable the communication of individuals through the environment. The adaptation of these mechanisms to an artificial system can be complex, mainly due to the absence of physical devices operating in a similarly way than the biological ones when detecting or emitting pheromones. We propose the use of quantitative sematectonic stigmergy, ie the deposit of the same individuals in the environment as a mechanism for communicating quantitative information.

This way, *beacon* individuals are a major players in this process. Their task is to receive virtual pheromones from nearby robots (within their communication range) and to store them. In addition, in order to emulate the operation of natural pheromones they are also responsible for decreasing the stored pheromones intensity as time goes by. This provides a good *forgetting* mechanism that is very useful in dynamic environments.

In our behaviour, the use of pheromones is essential and enables its effective development. The *wander* individuals emit pheromones around them when moving with a certain radius $\Psi_{r_{w}}\in R$ and intensity $\Psi_{\delta_{w}}\in R$. In the same way, in the transition between *wander* and *beacon* states, the vehicles will emit a pheromone footprint with larger radius $\Psi_{r_{b}}\in R$ and intensity $\Psi_{\delta_{b}}\in R$ than the previous one. This footprint is emitted only once, losing its intensity over time, where the rate for decreasing per second is indicated by the parameter $\Psi_{\in }\in R$.

The navigation of the individuals who are in the state *wander* is established by a potential field function **f**, determined by three components (**f**_1_, **f**_2_, **f**_3_). The movement to be developed by the robots is the sum of all three components

$$f=\overset{3}{\underset{i=1}{\sum }}\left(\alpha_{i}\cdot f_{i}\right)$$

where the intensity of each component is graduated with a specific factor *α*_*i*_. In order to develop this movement we are solely interested in the unit vector $\hat{f}=\frac{f}{\left|f\right|}$, since it is the one that indicates the direction to be taken.

The first component **f**_1_ is obtained as a vector pointing to the area of lower intensity of pheromones in the range of the vehicle, so that the importance of a detected pheromone decreases within the square of the distance. Assuming that the vehicle position is the origin of coordinates, we can specify this component as follows:

$$\begin{array}{c} C=\{\left(x,y\right)|\sqrt{x^{2}+y^{2}}\leq \Psi_{r_{b}}\} \end{array}$$

$$\begin{array}{c} f_{1}=\sum_{c\in C}(\frac{v(c_{y},c_{x})}{|v\left(c_{y},c_{x}\right){|}^{2}}\cdot \Psi_{field_{y,x}}) \end{array}$$

where *C* is a set of tuples with all the points that belong to the discretization of the circle (that depends on the resolution of the used sensor) within the pheromone radius *Ψ*_*r*_*b*__ and centered at robot position. **v**(*c*_*y*_, *c*_*x*_) is a vector which is constructed by the coordinates of the tuple (*x*, *y*) of each element of the set *C*. *Ψ*_*field*_*y*, *x*__ is the component (*y*, *x*) of a matrix containing the pheromone readings in the space coordinates (*x*, *y*) returned by the virtual stigmergy sensor.

The second component **f**_2_, determines the obstacle avoidance part. It is obtained by calculating a vector pointing to the area with fewer obstacles detected by the ultrasonic sensors:

$$\begin{array}{c} f_{2}=\sum_{j=1}^{\left|R\right|}r_{j}-p \end{array}$$

where *R* is the set of detected obstacles, **r**_**j**_ the position of the detected obstacle *j* and **p** the current position of the robot.

Finally, we will consider that the data perceived by sensors and sent to the actuators could contain inaccuracies, so a random component will be included in our selection process in order to make the next move taking into account the intrinsic uncertainty of robotic systems:

$$\begin{array}{c} f_{3}\left(t\right)=f_{3}\left(t-1\right)+\alpha_{4}\cdot rand\left(\right) \end{array}$$

Where *t* indicates current time, **rand**() is a uniform vector of random numbers, so that *rand*_*i*_ ∈ [0, 1] and *α*_4_ is a term that allows to adjust the importance of the random influence.

The provided microscopic behaviour is stochastic, as is deduced from its definition, and therefore small failures in perception or in actuators have no significant consequences. As has been presented, it includes intrinsically a noise term for the calculation of the trajectory to be followed by an agent that alter its movement trying to model real-world conditions.

In a more formal way, we define the microscopic behaviour to be developed by the individuals of the swarm as a tuple such that:

$$\begin{array}{c} \Theta =(\gamma ,\hat{f},\Psi ,\Gamma ) \end{array}$$

Where *γ* ∈ {*wander*, *beacon*} represents the current state of the individual and $\hat{f}$ sets the direction to be taken in the next time step. Ψ is a tuple that determines the characteristics of the stigmergy system, such that:

$$\begin{array}{c} \Psi =(\delta_{w},\delta_{b},\epsilon ,r_{w},r_{b},li) \end{array}$$

Where Ψ_*δ*_*w*__,Ψ_*δ*_*b*__ determines the intensity of emission of pheromones for states *wander* and *beacon* respectively. Ψ_*ϵ*_ defines the decreasing rate of virtual pheromones per second in the environment. Ψ_*r*_*w*__,Ψ_*r*_*b*__ establish the radius of pheromone emission in metres for states *wander* and *beacon*. *li* is the guaranteed maximum distance for stable communication between devices. It depends directly on the physical transceiver, the environment and the pheromones radius Ψ_*r*_*w*__,Ψ_*r*_*b*__. Finally, Γ is another tuple that includes all the common terms of the microscopic behaviour:

$$\begin{array}{c} \Gamma =(r_{u},\eta ,p_{0},\alpha_{1},\alpha_{2},\alpha_{3},\alpha_{4}) \end{array}$$

Where $r_{u}\in R$, is the radius of detection of obstacles for each vehicle, η∈R the rate of individuals to be added to the swarm per second, $p_{0}\in R^{2}$ is the initial position where behaviour will be developed and the drones will be launched and $\alpha_{i}\in R$ are the adjusting terms needed to obtain vector $\hat{f}$.

### Behaviour execution

Once the behaviour has been introduced, we will briefly discuss an example of execution. We will use a map within those presented in [30]. This map has a size of 100x100 metres.

**Fig 1:**
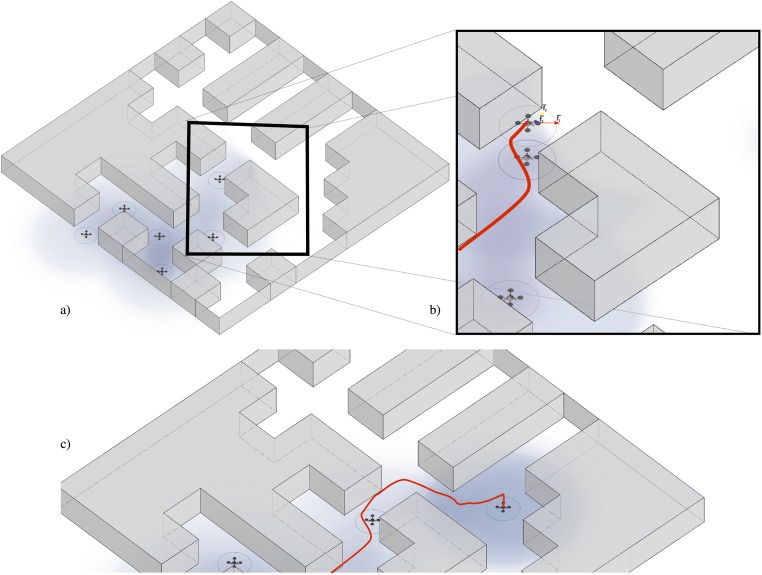
In a) we can see the 6th minute of the execution of the presented behavior, represented in a 3D map. This figure represents a part of the sequence presented in Fig 2 and it is chosen to illustrate the behaviour operation. In a) all vehicles are in beacon state (landed). This causes a new UAV to be introduced into the system from the starting position. This vehicle will traverse the environment following the indications of its speed vectors. In b) it is observed that the active (flying) UAV surpass the farthest landed vehicle (from the starting point). The 3 vectors that conform its speed (direction of the zone of less pheromones, obstacles repulsion, and slight random variation of the current speed) are observed. While moving, the UAV slightly modify the pheromone map by increasing it where it navigate. In c) the vehicle continues the flight until it finds that its quality of communication with the swarm decreases from the established thresholds and land, to form part of the group of repeating vehicles. When it lands it emits pheromones in its current position.

Initially, a robot in *beacon* state will be positioned at **p**_0_ = (35, 93). Starting from this position, new robots will be added progressively each minute. We can see the evolution of this behaviour from minute 5 to 8 in Fig 1. In this figure we could see a 3D representation of the environment and a detailed explanation of the presented behaviour.

**Fig 2:**
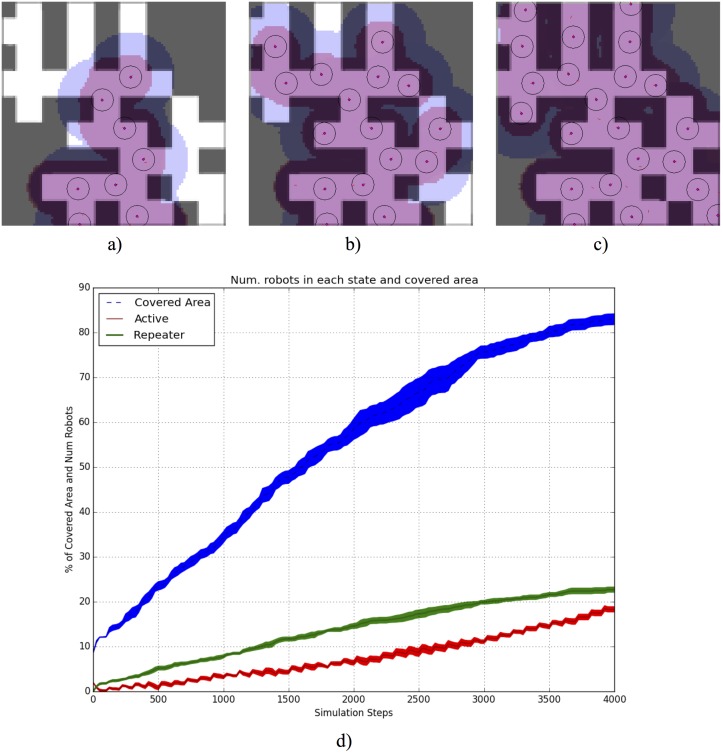
a) Covering progress of the microscopic behaviour, starting at position (35, 93), for 8’ of execution. Blue footprints represents the emitted pheromones. Red footprints represents the area of the environment covered by the swarm. b) Covering at 18‘. c) Covering at 30’. d) The mean and standard errors for both the number of individuals in each state of the simulation, and the number of individuals predicted by the macroscopic model are presented for 30 simulations. Robot position and obstacles detection range is shown for each robot. The percentage of area covered by the swarm is also showed, where each step is equal to 1/2 seconds.

In Fig 2 three later steps of the covering process (at 8, 18 and 30’ of execution) can be observed. In addition, a graph showing the mean and standard error of 30 simulations (using the same environment and parameters) is shown. This graph shows the percentage of covered area and the number of individuals in each state.

Therefore, the effectiveness of the environment covering process can be observed. It takes 30 minutes to cover the whole area. In the early stages of the behaviour the number of *beacon* agents increases quickly. This is because it is very easy for an individual to find an empty area not covered by any radio frequency signal used for stigmergy. As time progresses, the number of individuals in *wander* state increases since it is more difficult to find an uncovered area and new *wander* agents are being added.

## Macroscopic model

For the detailed analysis of the microscopic model it is essential to provide a mathematical model, able to analyze the global behaviour of the swarm in a given environment. The main reason for using a state-based behaviour is to allow the macroscopic model to be modelled as a recurrence equation taking into account the transitions between system states. Thereby, this macroscopic model can help us to predict the total number of individuals needed to cover an area of the environment, to adjust the radius of pheromone emission for optimal covering or even to make an estimate of the energy required by the swarm to develop their task.

The most important part to be analyzed in this behaviour is the transition between the two states of the system, *wander* and *beacon*. This is for example more valuable than getting the final distribution of individuals position, as this can be easily estimated by knowing the environment to be covered. Thus, we will model the number of agents found in each of the states of the system using the following system of recurrence equations:

$$\begin{array}{cc} w_{t} & =w_{t-1}-a_{t}\cdot w_{t-1}+\alpha_{5}\\ b_{t} & =a_{t}\cdot w_{t-1}+b_{t-1}\\ a_{t} & =\alpha_{6}\cdot a_{t-1} \end{array}$$

Where *w*_*t*_ represents the number of agents in *wander* state at time *t* and *b*_*t*_ the number of agents in *beacon* state at time *t*. Initially, in this behaviour it is much more likely that an individual turns its state from *wander* to *beacon*. This is because the environment is empty and therefore there is a larger area to be covered. Thus, function *a*_*t*_ allows to model the flow of individuals changing their state from *wander* to *beacon* through the initial value *a*_0_ and the term *α*_6_. In the previous equation, the number of agents to be added to the swarm is modelled in parameter *α*_5_. In our case this parameter takes a value of *α*_5_ = 0.01.

As it has been previously mentioned, the microscopic behaviour begins with an individual in *beacon* state and no one in *wander* state, so:

$$\begin{array}{c} w_{0}=0\\ b_{0}=1 \end{array}$$

### Obtaining *a*_0_ and *α*_6_

*α*_6_ ∈ [0, 1] determines how *a*_*t*_ decreases over time, which is directly related to the number of individuals in state *w* and *b*. A higher value of *α*_6_ will add more individuals to *beacon* state for longer. On the other hand, if its value is decreased the inclusion of individuals in *wander* state will be prioritized. This parameter is required to specify the fact that initially it is easy for individuals, because of the use of pheromones and the emptiness of the covering zone, to go very quickly to *beacon* state. As time passes this trend will decrease because more zones are covered and thus, more *wander* individuals will be seeking for free zones to land.

So, the maximum influence of this parameter is determined by the size of the radius of pheromones used in the model. A quadratic relationship between this radius Ψ_*r*_*b*__ and *α*_6_ exists, so that

$$\alpha_{6}=\lambda_{0}+\lambda_{1}\cdot \Psi_{r_{b}}+\lambda_{2}\cdot \Psi_{r_{b}}^{2}.$$

To calculate the following coefficients we have used a polynomial regression adjustment from various base models. This process can also be done automatically. As discussed in [39] it is not a complex task and can be done using learning techniques even for more complicated differential equations. More specifically, we obtain for our system that:

$$\{\begin{array}{l} \lambda_{0}=1\\ \lambda_{1}=2.5\cdot 10^{-5}\\ \lambda_{2}=2.5\cdot 10^{-6} \end{array}$$

Where Ψ_*r*_*b*__ is the radius of the pheromones in metres left by *beacon* individuals, as it has been previously seen in the description of the microscopic behaviour.

Moreover, *a*_0_ defines the ratio of initial individuals that will change to *beacon* state (and therefore will come out from *wander* state). This ratio depends mainly on two factors. On the one hand, the complexity of the environment and thus the distribution of walls or doors, influences in a negative way the navigation process required for the individuals for covering the environment. Of the other hand, seems reasonable to consider that in complex environments *a*_0_ decreases and therefore the existence of *wander* robots will be incremented. Furthermore, in larger environments with more empty areas where UAVs can fly, there will be more *beacon* individuals and parameter *a*_0_ must be increased.

This way:

$$\begin{array}{c} a_{0}=\lambda_{3}+\lambda_{4}\sum_{k=0}^{1}P_{k}log_{2}\left(P_{k}\right)+\lambda_{5}\cdot env_{size} \end{array}$$

Where $-\sum_{k=0}^{1}p_{k}log_{2}\left(P_{k}\right)$ refers to the complexity of the environment by calculating its entropy as a measure of reference. Thus, for a binary image representing free and occupied space we will obtain the probability of a pixel to represent it (term *P*_*k*_) where *k* is the grey level of the pixel (in the case of binary images, as in our case, we assume that images consist of occupied space *black* and empty space *white*). Moreover λ_5_ ⋅ *env*_*size*_ increases the value of *a*_0_ in a linear way. Where *env*_*size*_ is the environment size in square metres and λ_3_, λ_4_ y λ_5_ are adjustment terms.

The entropy of the maps is used as an indication of its navigability. There may be cases in which a map with more entropy is not more complex than another (large areas with small complex zones, such as a sequence of gates, may have a smaller entropy than small areas with irregular but highly navigable walls). Therefore, we assume uniformity of the entropy within the map, with similar areas distributed with the same density.

As in the previous case, it is easy to obtain an adjustment of the free parameters from several base models. For our system we have:

$$\{\begin{array}{l} \lambda_{3}=9.5\cdot 10^{-2}\\ \lambda_{4}=1/60\\ \lambda_{5}=5.4\cdot 10^{-6} \end{array}$$

## Experimentation

In this section, the behavioural functioning of the swarm will be shown, for simple, complex and unstructured maps. As discussed below, given the probabilistic nature of this system, several simulations for the same behaviour will be carried out for the development of the tests. Thus, a statistical analysis of the results will be presented.

Firstly, we will explore the influence of the artificial pheromones radius Ψ_*r*_*b*__ in the microscopic behaviour. In order to design a physical device for stigmergy, it is very important to analyse the importance of this radius because it is directly related to the evolution of behaviour and to the environment data required to be transmitted or stored in the swarm individuals.

Secondly, modelling mathematically this behaviour and analyzing its evolution is crucial. Therefore, in this section we will validate macroscopic model under consideration, analyzing the fitting error of the number of states prediction with our simulation over time. In addition, the number of individuals in each state for behaviour completion will be shown for both simulation and prediction. In swarm robotics, knowing the macroscopic model helps us to make important decisions about the microscopic model parameters to be used.

A limited but representative set of maps of the usual flight areas for this type of vehicle have been used. Specifically, unobstructed areas (the most common in flights with sufficient altitude), interior areas or low-altitude exterior flights (made up of walls and obstacles) and unstructured areas (inclined walls, curves without geometric structure). All the tested maps have the following characteristics, needed for the proper functioning of the robotic swarm: firstly, the size of the navigable areas should be enough so that navigation and avoidance of obstacles is developed effectively given the speed of the aircraft. Secondly, the entropy of the maps is used as an indication of its navigability. As commented before, we assume uniformity of the entropy within the map, with similar areas distributed with the same density.

### Pheromone radius Ψ_*r*_*b*__

Although there are many parameters that can be studied, the radius of pheromones takes special importance for this behaviour. This is a distinguishing feature that determines not only the distance between swarm individuals, but as discussed below, the average speed to complete the covering process. The microscopic behaviour that will be used in our tests is:

$$\left\{ \begin{array}{cc} \Theta & =(\gamma ,\hat{f},\Psi ,\Gamma )\\ \Psi & =(0.03,\frac{swarm_{size}-robot_{Id}}{swarm_{size}},0.0001,r_{w},r_{b},r_{b})\\ \Gamma & =(5,5,0.01,(5,80),0.2,0.4,0.4,0.4) \end{array}\right.$$

Where both *γ* and $\hat{f}$ do not vary from previous specification of the microscopic model. Moreover, Ψ_*δ*_*b*__ is defined from the robotic index (*robot*_*Id*_). This index starts from 0 and is incremented by one every time an individual is added. *swarm*_*size*_ is the maximum size of individuals that belong to the swarm. As the robots move away from the point of origin, the intensity of emission of pheromones in beacon state is lower (to encourage searching distant areas). That is why the agents of the swarm have an incremental index, that goes from 0 to the number of agents of the swarm minus one. Using this index, the intensity of emission of pheromones in beacon mode is obtained, so that the first agents introduced will be the one that will emit more intensity as stated in Ψ_*δ*_*b*__

The same launch position is used Γ_*p*_0__ in order to simplify testing in all maps, although in our experiments we have verified that this position does not substantially vary the result of the final covering.

The first test we perform determines the influence of the pheromones ratio at final covering state and the amount of time required. We have analyzed 4 different Ψ_*r*_*b*__ radios (10, 20, 30 and 40m), where $\Psi_{r_{w}}=\frac{\Psi_{r_{b}}}{2}$. We will assume that *li* depends directly on the communication range that is able to reach our pheromone system and therefore *li* = Ψ_*r*_*b*__. In this test, 30 runs were executed for each pheromone radius for a total of 10 maps (of different sizes and complexities). The results obtained for 4 selected maps are shown on Table 1.

**Table 1:** Selection of results for four maps of the simulation. All maps are delimited with perimeter walls. The first map (Id 1) is an empty hall of 100x100m (left map of Fig 3a). Map 2 is a 100x200m empty hall. Map 3 is half divided hall with one wall and only a pass door. Map 4 is a complex 100x100m hall, with multiple walls and corridors (middle right map of Fig 3a). The entropy for each map ordered by increasing Id is (0.2405, 0.0027, 0.2817, 0.4639). The average of area covered for 10 runs and the average of time required to cover this area is shown. The maximum time allowed for simulation is 15000s. This time will be shorter if the swarm cover (using the *c*_*a*_ metric) the 95% of the area. Furthermore, the number of individuals in states *wander* and *beacon* *w*, *b* for the simulation and for the mathematical model $\left(\hat{w},\hat{b}\right)$ is presented. Two different covering metrics are shown: *c*_*a*_, the percentage of terrain that is covered by any individual of the swarm in any time and *c*_*b*_, that indicates the percentage of area covered by individuals in the final iteration of the behaviour, assuming a detection range of 10m per individual. Finally, the measure of the differences between the number of individuals in each state predicted and simulated (using root-mean-square deviation, RMSD) is also presented.

Id	*φ*_*r*_	w	b	$\hat{w}$	$\hat{b}$	t(s)	*c*_*a*_	*c*_*b*_	*rmsd*_*w*_	*rmsd*_*b*_
1	10	5.60	233.40	0.50	237.99	11875	96.75	100.00	2.772	2.458
1	20	1.20	27.80	1.85	27.14	1400	94.03	14.80	0.4869	0.6688
1	30	3.20	12.80	3.13	12.86	750	92.44	8.16	0.5095	0.6684
1	40	7.80	8.20	7.33	8.41	738	93.96	8.16	0.6403	0.7511
2	10	8.00	293.00	8.82	292.17	15000	98.63	100.00	2.293	1.967
2	20	3.20	57.80	2.00	58.74	2988	93.38	31.12	1.162	0.8114
2	30	4.20	23.80	3.76	23.98	1338	92.99	14.29	0.7449	0.4387
2	40	7.20	14.80	8.38	13.61	1050	92.01	11.22	0.8336	0.7567
3	10	41.00	247.00	58.66	228.83	14325	95.87	100.00	5.13	5.075
3	20	15.20	27.80	15.70	26.54	2062	88.06	21.94	1.016	0.937
3	30	10.20	11.80	8.51	13.48	1050	89.06	11.22	1.33	1.066
3	40	15.20	7.80	15.43	7.31	1088	94.28	11.73	0.6473	0.4651
4	10	59.00	242.00	63.69	237.30	15000	95.33	100.00	5.999	5.716
4	20	40.40	30.60	42.88	27.61	3475	91.35	36.22	1.595	1.386
4	30	31.00	13.00	31.60	11.89	2125	93.20	22.45	0.7561	0.5267
4	40	16.00	7.00	17.04	5.45	1075	87.27	11.73	0.9312	1.199

For coverage analysis we have used two metrics. *c*_*a*_ represents the percentage of area covered by each individual at any time, ie, it is assumed that an individual has memory and can store the necessary environmental data to be transmitted while it is in *wander* state. *c*_*b*_ indicates the percentage of area covered by individuals in the final iteration of the behaviour, assuming a detection range of around 10m per robot. This is the opposite case, where the individuals have no memory of their navigation while staying in wander state and can only transmit information about the environment in *beacon* state.

The effect of increasing the pheromones radio Ψ_*r*_*b*__ (and indirectly Ψ_*r*_*w*__) is the distance between individuals in the final step of the coverage. With small values the covering is made much denser, reaching values of coverage close to 100%. However, as shown in Table 1, the time and the number of agents required for the deployment is much higher.

If the swarm individuals can have some type of memory that allows them to remember the last walked areas, the density of the deployment can be substantially reduced. In this case, a larger radius of pheromones have lower temporary cost at the expense of a more spaced covering, thereby *c*_*b*_ is substantially reduced. However *c*_*a*_ remains within acceptable levels, since the movement of the wander agents (and the area covered while moving) is taken into account.

We must not forget that if the individuals are not able to store the necessary information from the environment while moving (eg they are not able to locate where the readings are taken, which is far from being a trivial problem), the final position of the individuals in the behaviour is fundamental. In this case, as presented in Table 1 the pheromones radius used must be determined by two factors, *c*_*b*_, that decreases substantially at higher pheromones ratio Ψ_*r*_*b*__ and the time required for deployment which is increased by this radius. Also, it is important to underline that in the case of UAVs, the energy required when most agents are in state *wander* is significant. So depending on the environment, may not be feasible to increase the pheromones radius due to the quantity of energy required to maintain multiple agents in flight simultaneously.

**Fig 3:**
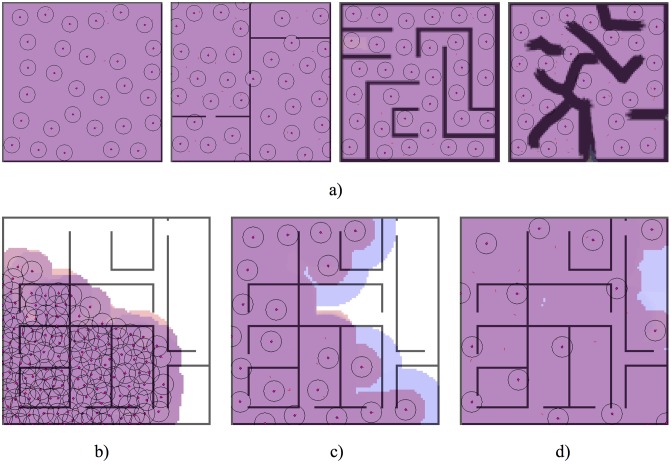
a) Environment covering results using the proposed microscopic behaviour with the default parameters and a pheromone radius of 20m. This behaviour terminates when *c*_*a*_ ≥ 95%. As previous figures red footprints represents the covered space (using *c*_*a*_ metric) and blue footprints defines the zones where the pheromones has been emitted. b) Status of the simulation at time 8144s with a pheromone radius of 10m. c) Status of the simulation at time 1700s with a pheromone radius of 20m. d) Status of the simulation at 2000s with a pheromone radius of 30m.

In Fig 3 an example of the result of coverage of this behaviour is presented for different maps and pheromone radius Ψ_*r*_*b*__. In Fig 3a the radio is constant at 20m. As we can see, the behaviour is functional regardless of the nature of the environment. In Fig 3b the same environment is used with different pheromones radius Ψ_*r*_*b*__ between 10, 20 and 30m. As it can be observed the difference when altering this parameter is the density of agents required to complete the coverage.

### Microscopic vs macroscopic model

Furthermore it is essential to verify whether our macroscopic model effectively models our achieved experimental results. To do so, we compare the prediction of the theoretical model by using the same experimental data in the previous section. Specifically, the macroscopic model is defined by the following parameters:

$$\left\{ \begin{array}{c} \lambda_{0}=1\\ \lambda_{1}=2.5\cdot 10^{-5}\\ \lambda_{2}=2.5\cdot 10^{-6}\\ \lambda_{3}=9.5\cdot 10^{-2}\\ \lambda_{4}=1/60\\ \lambda_{5}=5.4\cdot 10^{-6} \end{array}\right.$$

In the last two columns of Table 1 the root-mean-square deviation of variables *w*, *b* (individuals in *wander* and *beacon* state respectively) is presented. It is a frequently used measure of the difference between values predicted by a model or an estimator and the values actually observed.

Given the 10 analyzed maps, where their entropy varies from 0 to 0.7 and their size from 100x100mm to 400x400mm, we have obtained the following adjustment results presented in Table 2.

**Table 2:** Mean and standard deviation of *beacon* and *wander* RMSD (comparing the number of individuals in each state in simulation vs macroscopic predicted values) for 10 analyzed maps. The entropy of these maps varies from 0 to 0.7 and their size from 100x100mm to 400x400mm.

mean *rmsd*_*w*_	std *rmsd*_*w*_	mean *rmsd*_*b*_	std *rmsd*_*b*_
1.6429	1.4356	1.5501	1.4677

As it can be seen, with this values a good rate of adjustment of the microscopic and macroscopic model is achieved. In addition, in Table 1 the final number of individuals needed for the covering process could be observed for both, the simulated and the predicted model. The differences between them are not significant and, in this way, our macroscopic model could be used to predict the features needed by the microscopic model to deploy and cover and environment in an optimal way. More specifically, we have selected two cases for the analysis of the model adjustment to microscopic data.

**Fig 4:**
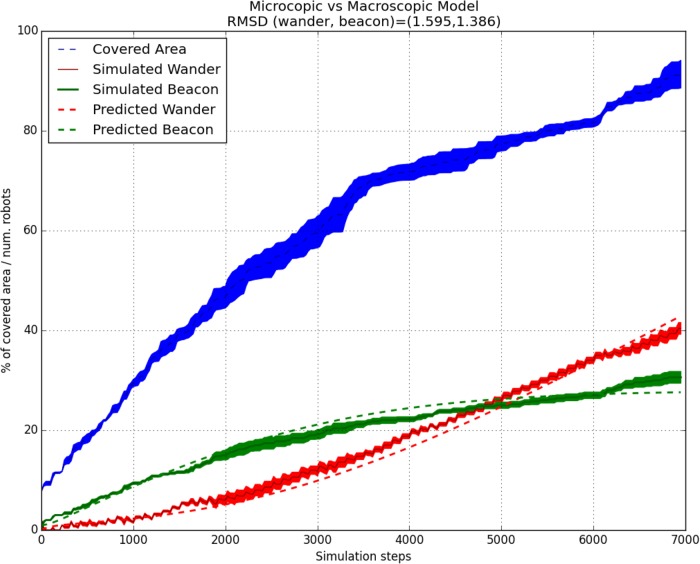
Comparison of the microscopic model with the macroscopic model for the labyrinth (center-right map) in Fig 3a, of size 100x100m and with pheromones radius of 20m. The mean and standard errors for both the number of individuals in each state of the simulation, and the number of individuals predicted by the macroscopic model are presented. In addition, the covered area *c*_*a*_ at each instant and the root-mean-square deviation of each state are presented. Each simulation step is equal to 1/2 seconds.

In Fig 4 the evolution of individuals in *wander* and *beacon* state (*w*, *b*) is presented for the labyrinth (center-right map) in Fig 3a and a pheromone radius of 20m. In this case we use a complex environment, where the displacement can be difficult in areas with multiple walls and doors. As it can be observed, the number of individuals in *beacon* state increase in areas close to the swarm deployment. However, as time passes these areas are covered and the swarm should explore new areas. On this instance, the number of *wander* agents grows until it outnumbers the *beacon* individuals. As it can be seen, the provided model correctly reflects this behaviour.

**Fig 5:**
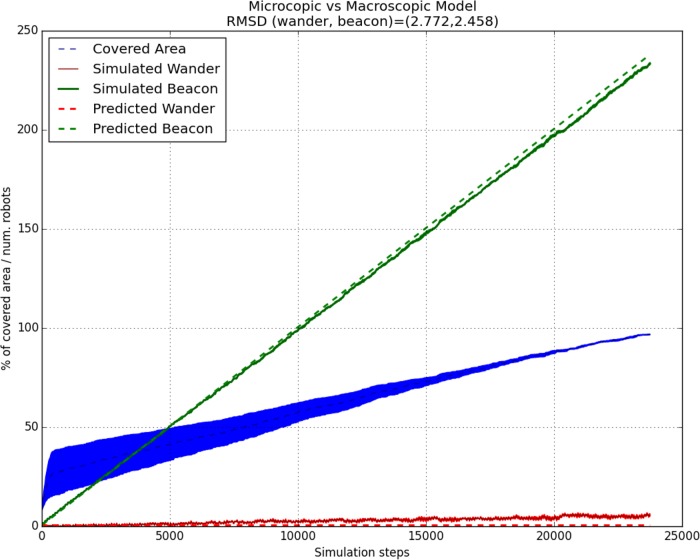
Comparison of the microscopic model with the macroscopic model for the most left map in Fig 3a, of size 100x100m and with pheromones radius of 10m. The mean and standard errors for both the number of individuals in each state of the simulation, and the number of individuals predicted by the macroscopic model are presented. In addition, the covered area *c*_*a*_ at each instant and the root-mean-square deviation of each state are presented. Each simulation step is equal to 1/2 seconds.

In Fig 5 the evolution of individuals in *wander* and *beacon* state (*w*, *b*) is presented for the left map in Fig 3a and a pheromone radius of 10m. This is a simple environment, without doors or buildings, where the deployment is produced progressively. Therefore, the number of individuals in state *beacon* is increased progressively, while only a constant number of *wander* individuals is used. Our microscopic model is able to correctly predict this case.

As has been commented before, we also use unstructured maps for these experiments obtaining good results in the simulations that have been carried out. The macroscopic model correctly predicts these environments without much variation with respect to structured maps. This result is expected, since no information regarding the structure of the environment is included in the microscopic model.

## Conclusions and future works

A microscopic model for a swarm behavior has been presented in this paper. As it has been seen, such model is able to deploy and cover an unknown area in a fully decentralized way using a virtual stigmergy system. This behaviour is adapted to the multi-rotor UAVs characteristics, establishing a power saving state for the individuals, taking into account the inherent actuators and sensors errors and providing a low computing load behaviour to be implemented on systems with limited computing capabilities, as it is often the case with this kind of vehicles.

This behaviour is oriented towards the implementation and use of the swarm within emergency situations, mainly due to the fact that it can be executed in any environment, without requiring the use of any existing communications networks or external localization services. Furthermore, this behaviour is able to create its own communication network for sending internal and external behavioural information and for obtaining environmental data to be analyzed.

In addition, a mathematical model for the macroscopic behaviour of the system has been provided. This model can accurately predict important behavioral data, such as the number of individuals needed to cover an area, the influence of pheromones radius, or the number of agents in each of the states of the system, allowing for example an analysis of the energy required by the swarm.

We have tested in an experimental way both, the influence of different radii of pheromones and the adjustment of the macroscopic model with the data obtained through simulation. For calculating the differences between the values predicted by the macroscopic model and the values actually observed in simulation we use the root-mean-square deviation (RMSD) measure, thus obtaining results that indicate that the presented microscopic model correctly models the behaviour of the swarm, regardless of the used environment. Therefore, we believe that this behaviour and model contributes and advances in the research line of swarm deployment and covering using UAVs.

Currently, two future lines of work are being taken into account. On the one hand we are developing a radio frequency physical sensor/emitter to use pheromones for real swarm of drones. This device can be used to perform indoor location tasks and to establish indirect communications of the swarm individuals. Therefore, it will provide a physical mechanism that directly supports stigmergy.

On the other hand, we are analyzing the energy required by the swarm by using our macroscopic model to determine the influence of the model parameters according to energy consumption. The main goal of this analysis is to obtain more efficient robotic swarms reducing their cost and increasing their autonomy. This type of systems can be applied for example to emergency and rescue tasks [40, 41], to create communication networks in restricted environments [42] or to search/rescue people or objects [43, 44].

## Supporting information

S1 File Simulation maps. Maps used in the simulation. Black pixels are not navigable. One pixel represent 1 meter in simulation.
(ZIP)

S2 File Data graphs. Original graphs that contains the simulation an model data.
(ZIP)
