# Supplementary figures and images for: Modelling multi-rotor UAVs swarm deployment using virtual pheromones
Source: PLoS One. 2018 Jan 25;13(1):e0190692. doi: 10.1371/journal.pone.0190692 (PMC5784900; doi:10.1371/journal.pone.0190692)

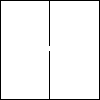

Supplement: S1 File — Maps used in the simulation. Black pixels are not navigable. One pixel represent 1 meter in simulation. (ZIP) [file pone.0190692.s001.zip › maps/S6_Fig.png]

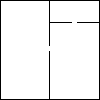

Supplement: S1 File — Maps used in the simulation. Black pixels are not navigable. One pixel represent 1 meter in simulation. (ZIP) [file pone.0190692.s001.zip › maps/S7_Fig.png]

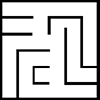

Supplement: S1 File — Maps used in the simulation. Black pixels are not navigable. One pixel represent 1 meter in simulation. (ZIP) [file pone.0190692.s001.zip › maps/S5_Fig.png]

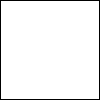

Supplement: S1 File — Maps used in the simulation. Black pixels are not navigable. One pixel represent 1 meter in simulation. (ZIP) [file pone.0190692.s001.zip › maps/S4_Fig.png]

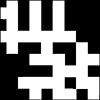

Supplement: S1 File — Maps used in the simulation. Black pixels are not navigable. One pixel represent 1 meter in simulation. (ZIP) [file pone.0190692.s001.zip › maps/S1_Fig.png]

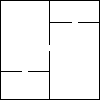

Supplement: S1 File — Maps used in the simulation. Black pixels are not navigable. One pixel represent 1 meter in simulation. (ZIP) [file pone.0190692.s001.zip › maps/S8_Fig.png]

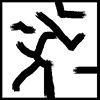

Supplement: S1 File — Maps used in the simulation. Black pixels are not navigable. One pixel represent 1 meter in simulation. (ZIP) [file pone.0190692.s001.zip › maps/S9_Fig.png]

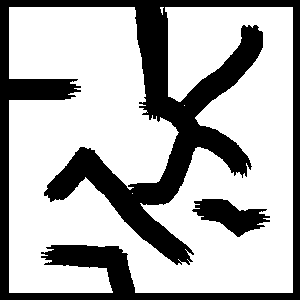

Supplement: S1 File — Maps used in the simulation. Black pixels are not navigable. One pixel represent 1 meter in simulation. (ZIP) [file pone.0190692.s001.zip › maps/S10_Fig.png]

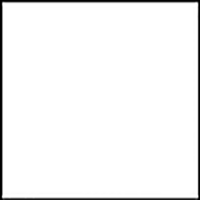

Supplement: S1 File — Maps used in the simulation. Black pixels are not navigable. One pixel represent 1 meter in simulation. (ZIP) [file pone.0190692.s001.zip › maps/S2_Fig.png]

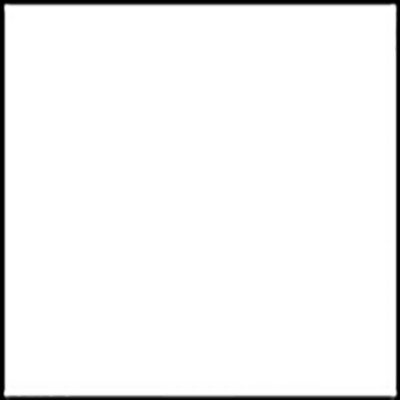

Supplement: S1 File — Maps used in the simulation. Black pixels are not navigable. One pixel represent 1 meter in simulation. (ZIP) [file pone.0190692.s001.zip › maps/S3_Fig.png]

Microcopic vs Macroscopic Model  
RMSD (wander, beacon)=(1.595,1.386)

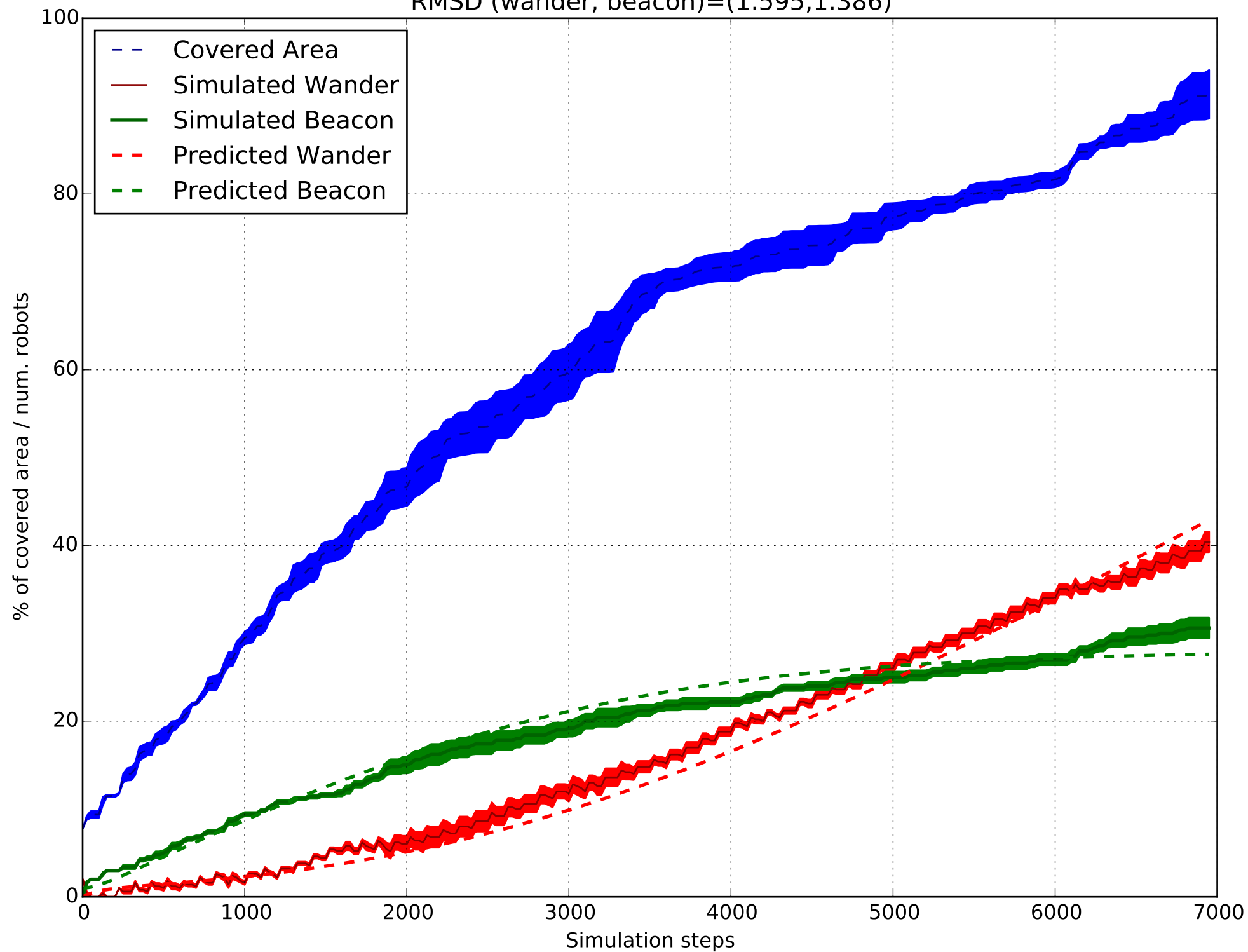

Supplement: S2 File — Original graphs that contains the simulation an model data. (ZIP) [file pone.0190692.s002.zip › graphs/S2_File.pdf]

Microscopic vs Macroscopic Model  
RMSD (wander, beacon)=(2.772,2.458)

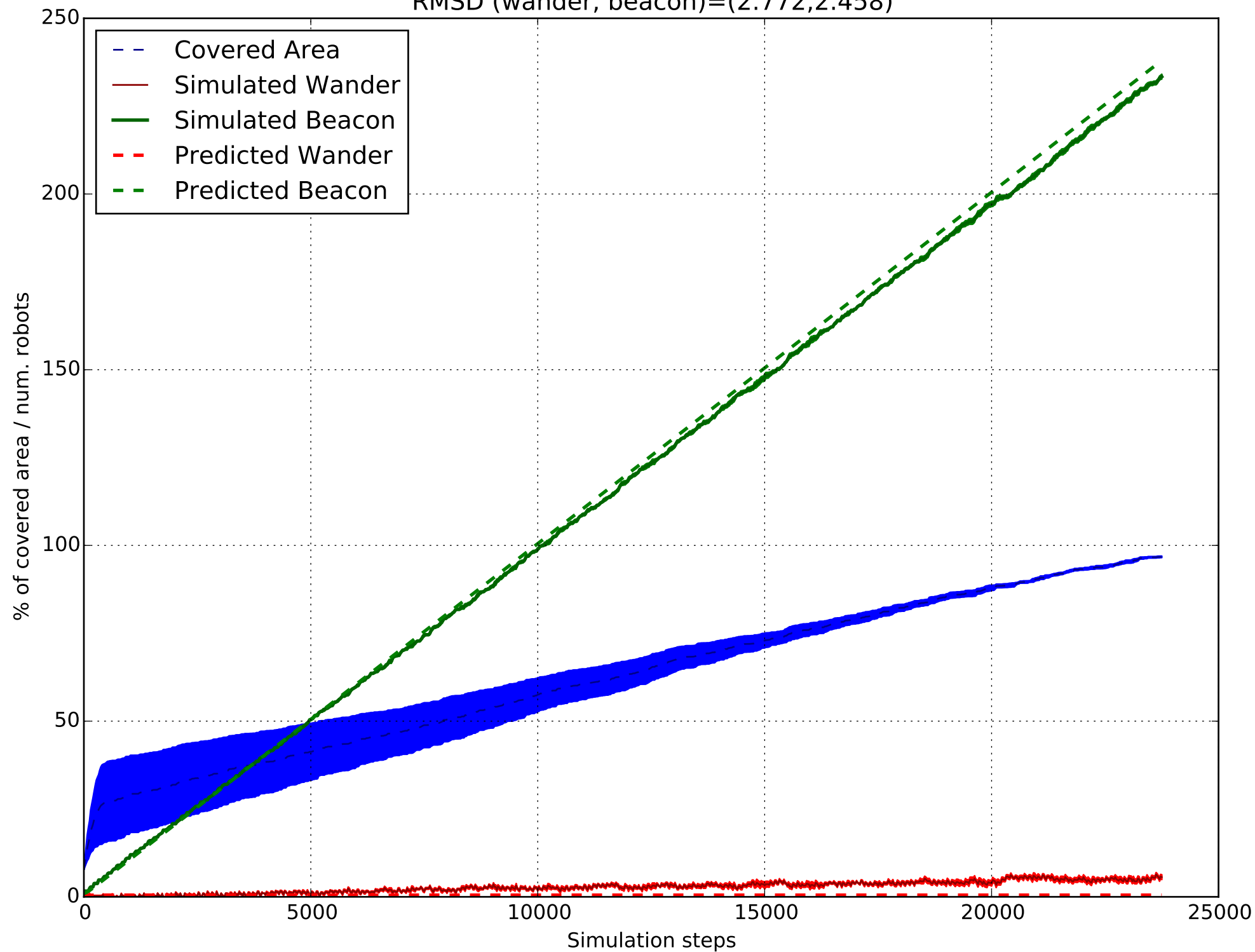

Supplement: S2 File — Original graphs that contains the simulation an model data. (ZIP) [file pone.0190692.s002.zip › graphs/S1_File.pdf]
